# Learning through a virtual patient vs. recorded lecture: a comparison of knowledge retention in a trauma case

**DOI:** 10.5116/ijme.5aa3.ccf2

**Published:** 2018-03-28

**Authors:** Olivier Courteille, Madelen Fahlstedt, Johnson Ho, Leif Hedman, Uno Fors, Hans von Holst, Li Felländer-Tsai, Hans Möller

**Affiliations:** 1 Department of Clinical Neuroscience, Karolinska Institutet, Stockholm, Sweden; 2 Unit of Neuronic Engineering, School of Technology and Health, Royal Institute of Technology, Huddinge, Stockholm, Sweden; 3 Department of Psychology, Umeå University, Umeå, Sweden; 4 Department of Computer and Systems Sciences, Stockholm University, Stockholm, Sweden; 5 Department of Clinical Science, Intervention and Technology, Division of Orthopaedics and Biotechnology, Karolin-ska Institutet, Karolinska University Hospital, Huddinge, Stockholm, Sweden

**Keywords:** Simulation-based trauma education, virtual patient, knowledge retention, biomechanics, Sweden

## Abstract

**Objectives:**

To compare medical students’ and residents’ knowledge retention of assessment, diagnosis and treatment procedures, as well as a learning experience, of patients with spinal trauma after training with either a Virtual Patient case or a video-recorded traditional lecture.

**Methods:**

A total of 170 volunteers (85 medical students and 85 residents in orthopedic surgery) were randomly allocated (stratified for student/resident and gender) to either a video-recorded standard lecture or a Virtual Patient-based training session where they interactively assessed a clinical case portraying a motorcycle accident. The knowledge retention was assessed by a test immediately following the educational intervention and repeated after a minimum of 2 months. Participants’ learning experiences were evaluated with exit questionnaires. A repeated-measures analysis of variance was applied on knowledge scores. A total of 81% (n = 138) of the participants completed both tests.

**Results:**

There was a small but significant decline in first and second test results for both groups (F_(1, 135)_ = 18.154, p = 0.00). However, no significant differences in short-term and long-term knowledge retention were observed between the two teaching methods. The Virtual Patient group reported higher learning experience levels in engagement, stimulation, general perception, and expectations.

**Conclusions:**

Participants’ levels engagement were reported in favor of the VP format. Similar knowledge retention was achieved through either a Virtual Patient or a recorded lecture.

## Introduction

Training future medical professionals means training a generation of digital natives who are accustomed to easily searchable information and who have quick global access to an increasing amount of open online educational programs, e.g., MOOCs (Massive Open Online Courses).^1^ Along with advances in Internet and computer technology, new challenging and interactive teaching methods, like e-learning and simulation-based learning, have been developed and implemented in medical education.^2^^,^^3^ However, the learning benefits of these new technologies are often overlooked and not always well evaluated by the stakeholders.^4^^,^^5^ Furthermore, little is known about their effectiveness in terms of knowledge understanding and retention.^6^^-^^8^

Research on simulation and gaming in higher education has shown that experiential learning through engaging and interactive simulation and authentic problem-solving situations improves knowledge acquisition and skills transfer by enabling recontextualization of acquired knowledge into practice, which is beneficial for learning outcomes.^9^^,^^10^ Hence, active learning environments contribute to the development of high-level thinking processes. However, knowing that long-term retention is critical for reinforcement of knowledge, it is also important to investigate if interactive training has the potential to lead to positive short-term and long-term retention effects.

Correct assessment of patients with spinal trauma is often complex and requires a biomechanical perspective, which can both be difficult to visualize and to understand by students and residents. As reported by Botezatu and colleagues, Virtual Patients (VPs) have proven to engage and motivate trainees, as well as improve learning acquisition and understanding.^11^ It was therefore suggested to use the VP model to offer a valid learning alternative to traditional lecturing in spinal trauma. We thus hypothesized that a VP-driven simulation would lead to similar short-term and long-term effects on knowledge retention as traditional lecturing.

A trauma case targeting assessment, diagnosis, and treatment procedures of patients with spinal trauma was designed to test this hypothesis. A VP-based learning component was developed featuring interactive illness history and utilizing physical exams and lab/imaging tests, as well as three-dimensional visualizations of cervical spine injuries. The visualization modality used in the present learning model has previously shown face validity and demonstrated increased self-efficacy in medical students after such training.^12^^,^^13^

This study aimed to determine if medical students’ and residents’ knowledge retention in assessment, diagnosis, and treatment of trauma surgery cases after VP training would be on par with traditional lecturing on the same subject. The primary objective of this study was thus to compare the levels of knowledge acquisition (short-term retention) and knowledge decay (long-term retention) between a VP training session and a video-recorded traditional lecture of a spinal trauma case.

The secondary objective was to evaluate the potential educational benefits of the virtual learning environment in relation to traditional lecturing by assessing the participants’ appraisals (self-reported learning experience) of both teaching formats.

## Methods

### Study design and participants

**Figure 1:**
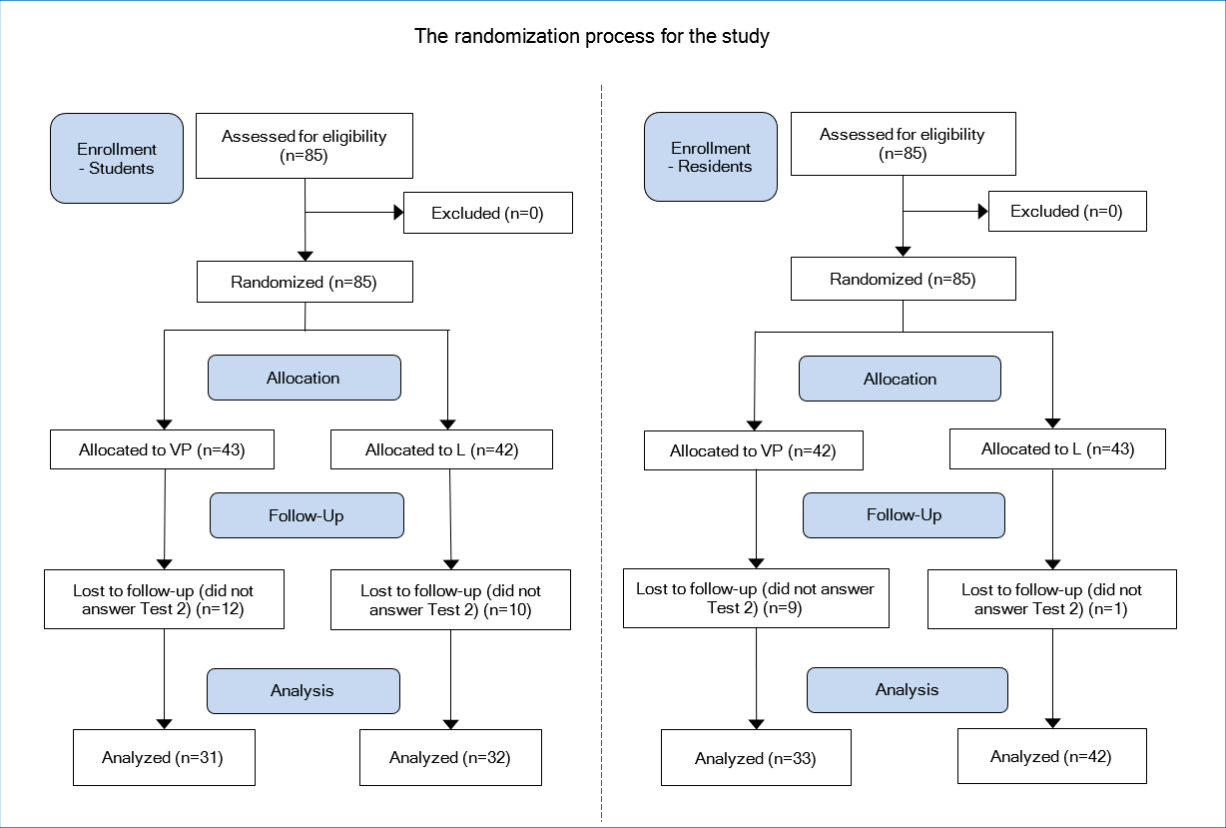
Randomization and allocation process (VP=Virtual patient session; L=Lecture session)

To compare the knowledge retention in the two intervention groups (a VP versus a videotaped traditional lecture (L)), the participants took a knowledge test just after they went through the intervention and then took another test at least two months after the intervention. The participants were 4th-year medical students at the Karolinska Institutet, Stockholm, Sweden, and orthopedic residents from national board courses held by the Orthopaedic Department at the Karolinska University Hospital, Stockholm, Sweden. The cohort was randomized into the two parallel teaching formats (VP and L) and was stratified for gender and educational level (medical student or resident). Participation in the study was on a voluntary basis and was offered as a complementary learning activity during courses in trauma management and spine surgery. The Regional Ethical Review Board approved the study in Stockholm. One hundred seventy persons participated in this study. Half of the participants were medical students with a mean age of 25.8 (SD 4.2) years, and the other half were residents in orthopedics with a mean age of 34.4 (SD 3.9) years. In the student group, 51 persons were females and 34 were males. For the residents, the distribution was 26 females and 59 males. The process of randomization, allocation, and follow up are described in Figure 1. Both knowledge tests (Test 1 and Test 2) were taken by 74% of the students and 88% of the residents, respectively (81% altogether, n=138). The mean number of days between the first and second knowledge test was 134 days and 117 days for students and residents, respectively.

### Intervention

All participants were first given a short introduction about the study and asked to sign an informed consent form. Each participant then went through his/her assigned teaching format individually. Both teaching formats focused on a cervical spine trauma case involving a man who was injured in a motorcycle accident and sustained fractures to the first and fifth cervical vertebra.

The lecture group watched a 13-minute video-recorded lecture combining PowerPoint slides and a filmed presenter (Picture in Picture). The participants were assigned an individual computer with headphones and watched the video immediately after the short introduction. The lecture included a discussion about evaluation and treatment of cervical spine fractures based on the presented trauma case. The lecture also featured a demonstration of accident reconstruction from a biomechanical perspective. The visualization was performed with a detailed three-dimensional finite-element model of the cervical spine developed at the KTH Royal Institute of Technology that included dynamic imaging sequences designed to enhance the understanding of the biomechanics behind an injury.^14^ The model consists of the vertebrae, ligaments, facet joint, discs, muscles, and skull. In the simulations, the kinematics and stress distribution was visualized at the time of impact. Prior to the three-dimensional simulations, a short introduction to the finite-element method was also given. The visualization with the finite-element neck model has been evaluated in a previous study by Hedman and colleagues, where it was judged by the students to be a good interactive scenario-based educational tool.^13^

The VP teaching format was based on the same trauma case. Participants were instructed to act as the doctor assessing the patient at an emergency unit. First, they had the chance to ask the VP medical-history questions with different degrees of relevance for the case. After taking the history, the participant was able to move on and examine the patient and order laboratory or imaging tests.

After the examination, the participant was automatically introduced to the finite-element method and biomechanical simulations, as described for the L group, but interactively. The subsequent section focused on the mandatory assessment where the participant was asked to make a diagnosis, report to a senior doctor using the situational briefing tool SBAR (Situation Background Assessment Recommendation) for communication, and then discuss advantages and disadvantages regarding possible treatments.^15^ The whole session ended with summative feedback based on all of the participant’s actions and decisions during the whole VP interaction. On average, the session was completed in 45 minutes.

### Data collection

The primary outcome measure of the study was long-term knowledge retention. Therefore, the participants were instructed to take a knowledge test (“Test 1”, to control for short-term retention) directly after going through the training, and they were then given a post-test (“Test 2”, to control for long-term retention) after a minimum of 2 months. Both tests consisted of 12 multiple-choice questions. Four of the 12 questions had multiple correct answers. A correct answer gave one point for each question. The questions with multiple correct answers were given a full point when all the answers were correct, but only the ratio of the number of correct answers divided by the total amount of correct answer when the answer was partly correct. The same questions and choices were found in both tests, but the order of both the questions and the choices in Test 2 were purposely altered. The score of the tests was only included in the analysis if the participants had answered both tests.

The secondary outcome of the study was the participants’ self-reported learning experience with their allocated learning method. Directly after Test 1, participants were instructed to fill in a questionnaire asking about their attitudes toward and perceptions of the learning experience. The questionnaires for the VP group and the L group differed in length with more design specific questions for the VP group. Both groups were asked to fill in four closed-questions concerning IT experience and proficiency on 5-point Likert-type scales. They then answered 15 closed-questions concerning current cognitive and affective states (after the learning experience) on a 5-point Likert-type scale ranging from strongly disagree to strongly agree. The VP group answered two additional questions due to the distinctive design of the VP case featuring direct feedback. For both groups, the questionnaires ended with six questions concerning general opinions and perceptions about the learning experience (three open-ended questions and three closed-questions on 5-point Likert-type scales). Additionally, the VP group answered six extra (design-related) questions, including three open-ended questions and three closed-questions on 5-point Likert-type scales.

The self-reported questions on IT proficiency as well as cognitive and affective states were based on previous studies on engagement conducted by Hedman and colleagues, and Sharafi and colleagues.^16^^,^^17^ The remaining questions (opinions and perception) were defined and tested during a pilot study conducted one year earlier with 15 respondents indicating good construct validity with a satisfactory response range on 5-points Likert-type scales. All questionnaires were analyzed regardless of whether the participants answered Test 2 or not.

### Statistical procedures

To control for knowledge decay and retention, we applied a non-inferiority hypothesis (i.e., that the VP group would achieve similar levels of long-term retention as the L group). A non-inferiority margin of 1 point out of a maximum of 12 points (8.3%) in the total score of the knowledge test was chosen as a cut-off point by the research team. The sample size calculation gave a minimum requirement of 64 participants per group (residents, students) to detect a relative reduction in test scoring of more than one point in knowledge decay over time when using an alpha of 0.05 and 80% power. Given that 20% loss to follow up, 80 participants were required per group, making for a total sample size of 160 study participants. A repeated-measures analysis of variance was applied to knowledge scores (Test 1 and Test 2) using Statistica 12 (StatSoft).

## Results

### Knowledge acquisition and retention data

**Figure 2:**
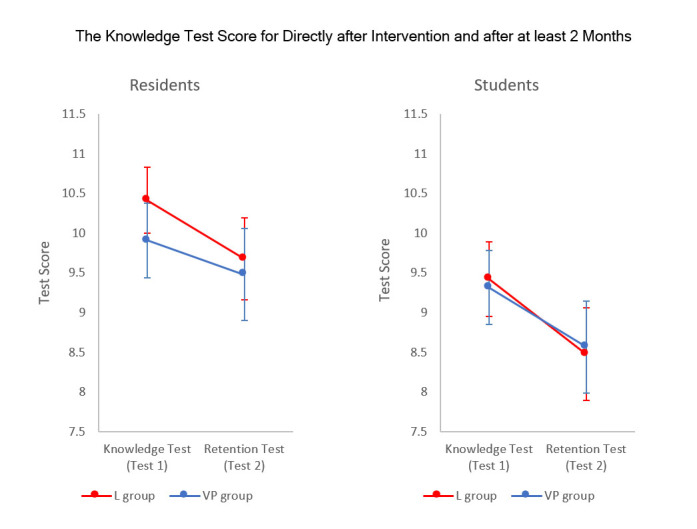
The results from the two tests (Test 1 – Knowledge test and Test 2- Retention test) for the two interventions (VP and L) and the two study groups (residents (left) and medical students (right)). Graphs are showing the mean values and 0.95 confidence intervals.(VP students n=31, L Students n=32, VP Residents n=33, L Residents n=42)

The repeated-measures analysis of variance showed that there was no significant difference in the knowledge acquisition test (Test 1) between the VP and L groups, implying similar short-term retention in both groups. As shown in Figure 2, a small but significant decline in knowledge retention over time (time factor from Test 1 to Test 2, F _(1, 135)_ = 18.154, p = 0.00) was observed in both groups. However, no significant differences in the long-term retention test (Test 2) were observed between the two groups.

### Learning experience

**Figure 3:**
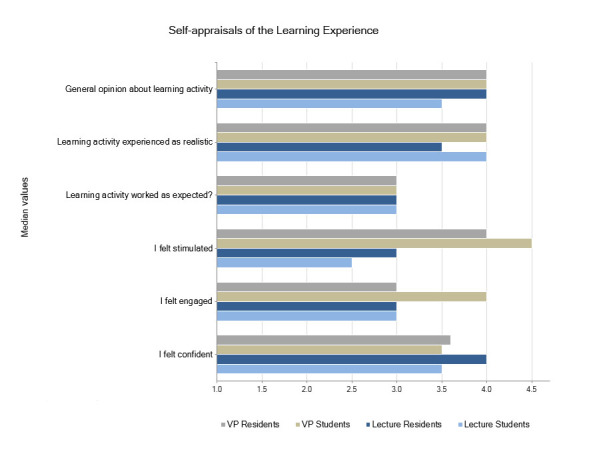
Likert-scale-based values regarding the appraisals to the Learning Experience Questionnaire

The participants’ self-reported learning experience is summarized in Figure 3. In general, there were no large discrepancies between the VP and L groups. The level of engagement, stimulation, general opinion, and learning expectations was reported with high median values.

Participants’ appraisals supported the face validity of the web-based lecture to an open-ended question about their first impressions and comments about the learning experience. Some relevant quotes from participant appraisals read:

“It felt like a regular lesson with a teacher talking and PowerPoint slides” [male respondent, student 6] … “It was like a normal lecture, neither better nor worse” [female respondent, resident 26] … “A standard lecture similar to many others.” [male respondent, resident 4]

General opinions and perceptions about the VP learning experience were evaluated using open-ended questions (self-reported experience). In general, the VP group more frequently acknowledged the clinical relevance of the case compares to the lecture group.   Some typical quotes from participants’ appraisals about the VP component read:

“Very good introduction to the way of thinking around trauma patients, but also relevant in general. I felt that I got feedback that was useful” [male respondent, student 4] ... “I learned much more than I would have done by just reading or attending a lecture at the same time.” [female respondent, resident 21].…  “Definitely useful as a complement to traditional studying: combines skills, simulates reality, sets "goals," immediate feedback.” [male respondent, resident 22]

## Discussion

This study conducted an evaluation of knowledge retention over time in two teaching formats, a VP and a traditional lecture, both featuring a biomechanical simulation of cervical spine trauma. The results support our main hypothesis that a VP session was on par with a video-recorded teaching session featuring a traditional lecture regarding both short-term and long-term retention of knowledge. This aligns well with other findings of retention effects of virtual training in health education.^18^^-^^20^ Additionally, and as opposed to traditional lecturing, the VP learning format accommodates multiple learning styles and offers a more active involvement in procedural training, which is beneficial for clinical skills development.^2^^,^^4^^,^^10^^,^^26^ This, in turn, can contribute to further improvement in self-efficacy.

Participants’ self-appraisals of the learning experience were reported in favor of the VP format, thus confirming earlier studies on motivation and learning engagement with interactive learning methods.^11^^,^^21^^-^^23^ The medium level of self-reported confidence in the VP group might be explained by insufficient prior experience with virtual-learning environments (in particular VP-based learning). Moreover, none of the participants had previously trained with the presented VP format. An alternative explanation is that the VP-based training method was more demanding in terms of challenging one’s clinical knowledge and that the session included individual feedback where the participant could compare their own decisions with those from an expert.

The hours that medical doctors spend in the hospital have decreased due to working-hour regulations that have been implemented to prevent exhausted doctors and thus to increase patient safety. Consequently, students experiencefewer opportunities with clinical cases overall and with rare cases in particular.

This increases the importance of actively working with patient cases in clinical training and education. Hence, within the examined VP session, students were trained in communicating with the patient and in performing the relevant examinations.

Although this study included a large number of randomized participants, there are some limitations in the study. Due to educational and structural constraints, no potential knowledge gain between the first and second knowledge test could be monitored. Further, the second test was performed online with no control regarding possible access to external aids. The participants were asked not to use any aids and were only allowed to submit the test answers once. Admittedly, the study evaluated trainee performance in only one patient case, whereas broader experience with several cases presumably would have affected the outcomes in both knowledge retention and participants’ learning experience. We can even assume that more regular training with virtual trauma cases would possibly lead to superior results in long-term retention because earlier studies on VP cases in undergraduate medical education have indicated better results in knowledge acquisition compared to traditional training.^9^ Because of the rapid increase in the use of VP cases, future studies should focus on when and how VP-based educational formats are most effective and should take into account human factors like gender, learning styles, and IT competence. Hence, a follow-up study aiming to measure long-term retention over a longer period after the training intervention (more than six months for performing the post-test) would certainly bring more insight on long-term effects. Although not reported here but seen as a possible trend in our variance analysis, we suspect that there might be a stronger benefit in retention efficiency for male students, which is worth investigating further in a follow-up study. The mix of live clinical encounters, traditional teaching methods, and VP formats is a challenging subject for investigation.

Further, there is clearly a need for learning tools where structured clinical communication can also be trained. In this VP-based model, the participant was trained using the SBAR protocol, which offers benefits in terms of training in standardized communication between healthcare workers.

The VP framework can also be developed for multiple trainers and possibly for inter-professional learning.

Innovative teaching methods, including state of the art virtual learning environments and simulations, have gained popularity in the educational arena.^24^^,^^25^ Using VPs in teaching has the potential to be cost-effective by reducing the need for lecturing time, which has relevance both for basic training as well as for continuing medical education and lifelong learning. Nonetheless, systematic validation and assessment of these new tools must be paralleled with implementation to guarantee evidence-based learning in future physicians.^26^^-^^29^

## Conclusions

This study showed similar short-term and long-term knowledge retention between the VP session and the video-recorded teaching session featuring a traditional lecture. Participants’ engagement level was reported in favor of the VP format. This format could be developed for multiple trainers and possibly for inter-professional learning, since the VP’s interactive contextualization and visualization of the accident history, combined with the application of assessment tools and clinical procedures, might play an important role for learning. However, further work needs to be done to evaluate the use of VP format for inter-professional learning. The study supports the use of VP in medical education as complementing traditional teaching formats. Policy makers in medical education and training might find these conclusions relevant in defining new teaching strategies for improving training efficiency.

### Acknowledgments

This study was supported by research grants from the Swedish Knowledge Foundation and the Stockholm County Council. The authors gratefully acknowledge Dr Paul Gerdhem for his contribution as a lecturer in the video-recorded lecture and Magnus Backheden for help with statistics. The authors would also like to acknowledge Kate LeBoeuf and Matthew Hogg for reviewing the language of the manuscript.

### Conflict of Interest

The authors declare that they have no conflict of interest.
